# In vivo testing of novel vaccine prototypes against *Actinobacillus pleuropneumoniae*

**DOI:** 10.1186/s13567-017-0502-x

**Published:** 2018-01-09

**Authors:** Fabio Antenucci, Cyrielle Fougeroux, Alannah Deeney, Cathrine Ørskov, Andrew Rycroft, Peter Johannes Holst, Anders Miki Bojesen

**Affiliations:** 1 Department of Veterinary and Animal Sciences, University of Copenhagen, Stigbøjlen 4, 1870 Frb. C., 1-20, Building: 301, Copenhagen, Denmark; 2 Department of International Health, Immunology and Microbiology ISIM, University of Copenhagen, Øster Farigmagsgade 5, Bldg 22/23, 1014 København K, Copenhagen, Denmark; 3 Department of Pathology and Pathogen Biology, Royal Veterinary College, Hawkshead Lane, North Mymms, Hertfordshire, AL9 7TA UK; 4 Department of Biomedical Sciences, University of Copenhagen, Blegdamsvej 3, 2200 København N, 12.3, Building: 32, Copenhagen, Denmark

## Abstract

*Actinobacillus pleuropneumoniae* (*A. pleuropneumoniae*) is a Gram-negative bacterium that represents the main cause of porcine pleuropneumonia in pigs, causing significant economic losses to the livestock industry worldwide. *A. pleuropneumoniae*, as the majority of Gram-negative bacteria, excrete vesicles from its outer membrane (OM), accordingly defined as outer membrane vesicles (OMVs). Thanks to their antigenic similarity to the OM, OMVs have emerged as a promising tool in vaccinology. In this study we describe the in vivo testing of several vaccine prototypes for the prevention of infection by all known *A. pleuropneumoniae* serotypes. Previously identified vaccine candidates, the recombinant proteins ApfA and VacJ, administered individually or in various combinations with the OMVs, were employed as vaccination strategies. Our data show that the addition of the OMVs in the vaccine formulations significantly increased the specific IgG titer against both ApfA and VacJ in the immunized animals, confirming the previously postulated potential of the OMVs as adjuvant. Unfortunately, the antibody response raised did not translate into an effective protection against *A. pleuropneumoniae* infection, as none of the immunized groups following challenge showed a significantly lower degree of lesions than the controls. Interestingly, quite the opposite was true, as the animals with the highest IgG titers were also the ones bearing the most extensive lesions in their lungs. These results shed new light on *A. pleuropneumoniae* pathogenicity, suggesting that antibody-mediated cytotoxicity from the host immune response may play a central role in the development of the lesions typically associated with *A. pleuropneumoniae* infections.

## Introduction

*Actinobacillus pleuropneumoniae* (*A. pleuropneumoniae*) is a Gram-negative bacterium responsible for a serious respiratory disease affecting pigs and a cause of large economic losses in the pig industry [1, 2]. *A. pleuropneumoniae* is transmitted from pig to pig by aerosols or direct contact and causes clinical signs such as vomiting, diarrhea, respiratory distress and bloody discharge from the mouth and nose, which may be lethal [3]. A total of 16 different *A. pleuropneumoniae* serotypes have been reported worldwide, classified according to capsular antigens [4, 5]. Several serotypes contain strains that can cause severe symptoms, which makes it challenging to develop a broadly protective vaccine. Currently available vaccines against App can be divided in two categories: (i) vaccines based on inactivated whole-cell bacterins; (ii) vaccines based on Apx toxins, a set of pore-forming cytolysins (Apx I–IV) playing a central role in App pathogenesis [3, 6]. Bacterin-based vaccines have been shown to offer limited protection against infections by strains other than the ones used for vaccination [7, 8]. While vaccines based on inactivated Apx toxins (toxoids) are effective in reducing the morbidity associated with infection [9–12] yet unable to prevent colonisation of the lungs, their use pose a potential threat inducing infection by asymptomatic carriers [7, 11, 13]. Thus, it would be highly desirable to develop a novel vaccine offering protection against App colonisation and morbidity in a cross-serotype manner.

The poor specificity of the detection methods for *A. pleuropneumoniae* and the rapid progression of the infection frequently prompt widespread application of antibiotic treatment on the mere suspicion of an infection [14]. This contributes to the overuse of antibiotics, which promotes selection of resistant bacteria and may indirectly lead to the spread of antibiotic resistance genes through the food chain [15, 16]. Accordingly, an effective vaccine against *A. pleuropneumoniae*, which protects across the 16 serovars is urgently needed.

The pathogenesis of porcine pleuropneumonia is complex and currently divided in different stages: colonization, resistance to clearance and damage to host tissues (direct and indirect) [3, 17, 18]. Colonization is usually the fist step in the establishment of a bacterial infection, and several *A. pleuropneumoniae* virulence factors have been shown to be involved in adhesion to the host tissues and expressed in vivo [19, 20]. *A. pleuropneumoniae* cells also show the ability to avoid clearance by both innate and adaptive immune responses thanks to mechanisms such as antiphagocytic activity and antibody degradation [3, 6, 21]. Finally, the lesions typically associated with porcine pleuropneumonia are mostly caused by a combination of toxins and proteases secreted by the pathogen and inflammatory mediators released by activated phagocytes [3, 22, 23].

As previously mentioned, cross-serotype prevention of porcine pleuropneumoniae remains elusive, and successful control of the infection with vaccination may require more in-depth knowledge of the pathogenesis of the disease. It is thus important to clarify how the establishment of the infection affects the course of the disease and if, as suggested; acute lung damage developed in the first days is the defining features for the severity of the disease [24]. Moreover it would be informative to understand if the infection can be controlled on a systemic level or mostly on a local level, thus determining the most efficient route of immunization.

In this study we describe the adoption of three different vaccination strategies for the prevention of porcine pleuropneumonia and lung colonization by *A. pleuropneumoniae*. Different strategies were selected in order to offer to the host immune system the widest possible range of immunogens and alternative administration routes. The first strategy involved the utilization of previously selected cross-serotype immunogens [25], expressed as recombinant proteins. Briefly, the conserved immunogens were selected in silico on the basis of exposition and accessibility of the proteins on the bacterial outer membrane (OM), as well as gene conservation among serovars, as described in [25]. From these studies we selected the proteins Apfa and VacJ, a pilin and an OM lipoprotein, respectively, whose role in *A. pleuropneumoniae* virulence previously has been described [11, 26, 27]. The selection of ApfA and VacJ as immunogens offered the potential of targeting *A. pleuropneumoniae* cells adhesion, OM integrity and resistance to clearance by the host immune system.

The second strategy involved the utilization of the antigenic outer membrane vesicles (OMVs) [28]. OMVs possess a variety of biological functions, and most importantly usually exhibit an antigenic pattern similar to the one found on the bacterial OM [29, 30]. Accordingly, *A. pleuropneumoniae* OMVs have shown promising results in terms of immunogenicity, offering the prospect of providing a range of conserved antigens [31]. Furthermore, thanks to their non-live nature, OMVs can be employed in vaccine formulations without incurring in the usual risks associated with live vaccines [29]. Notably, both autologous and heterologous OMVs have been reported to function as an adjuvant for co-administered recombinant antigens [32–34], but more recently some controversy has emerged regarding the possibility of combining OMVs with other immunogens [35]. To verify the potential of OMVs as adjuvants, we decided to combine OMVs and recombinant proteins ApfA and VacJ as a third strategy of immunization.

One of the technical challenges during an infection trial is to ensure the thoroughly and equivalent dissemination of infectious particles through the groups of animals. As previously mentioned, one of the main transmission routes of *A. pleuropneumoniae* infections is by aerosol [36, 37]. To simulate as closely as possible natural transmission, we selected an aerosol chamber transmission model as dissemination procedure for our in vivo challenge. This system is ideally suited for a respiratory pathogen such *A. pleuropneumoniae*, and has already been tested successfully in several studies [38–40].

## Materials and methods

### Animal model

A total of 55 10-kg piglets (Landrace-Yorkshire-Duroc crossbred) were purchased from a commercial breeder, which was part of the Danish Specific-Pathogen-Free (SPF) breeding system (code red), which is the highest health level in the system. Briefly, code red SPF herds are tested monthly for App clinical signs and by a serum sample ELISA aiming at serotypes 1–10 and 12, respectively. Upon arrival the pigs were housed in the animal facilities at the Department of Veterinary and Animal Sciences, (Frederiksberg, Copenhagen, Denmark). Piglets were ear-tagged with a unique identification number and randomly distributed in four different rooms that could be further separated in three smaller pens, after vaccination. Experiments were started after allowing the pigs to acclimatize for a week. Daily care was provided by animal caretakers blinded to treatment groups. Experiments were approved by the Danish national animal experiments inspectorate (Dyreforsøgstilsynet), as stipulated in license number 2014-15-0201-00019. Pigs were checked for presence of *A. pleuropneumoniae* by plating nose swaps on MHF plates [Mueller–Hinton agar + 5% horse blood + 20 mg/L ß-nicotinamide dinucleotide (NAD); BioMérieux], before initiation of the experiment.

### Immunogen production

Recombinant proteins ApfA and VacJ, and OMVs were produced and isolated as described in [25]. Briefly, *apfA* and *vacJ* genes from *A. pleuropneumoniae* L20 (serotype 5b) were cloned and expressed in competent *Escherichia coli* (*E. coli*) BL21 (DE3) cells, and the resulting ApfA and VacJ proteins isolated by size exclusion chromatography as described in [41]. OMVs were produced from *A. pleuropneumoniae* MIDG2331 (serotype 8) cultures and isolated by hydrostatic filtration, as described in [25].

### Immunization

**Figure 1:**
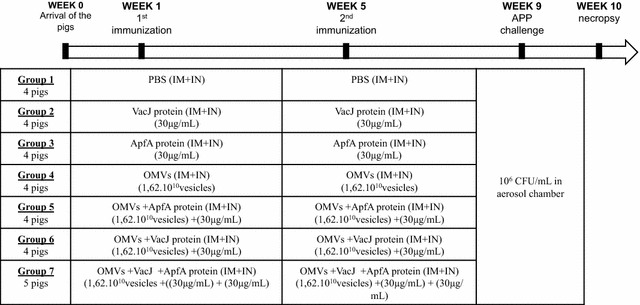
**Immunization and challenge regimen.** Immunizations were carried out intra-nasally with a Pasteur pipette and intra-muscularly by injection. Animals were immunized with either a single component or a combination of the following: 30 μg/mL protein (Apfa and/or VacJ), 1.62 × 10^10^ vesicles/mL OMV. All animals were re-immunized (boosted) 4 weeks after the first immunization. Finally, all pigs were challenged 8 weeks after the first immunization with live *A. pleuropneumoniae* HK361 via an aerosol chamber. A week after challenge, pigs were euthanized and subjected to necropsy.

The immunization and challenge procedure is summarized in Figure 1. After 1-week of acclimatization pigs were vaccinated intra-muscularly and intranasally either with OMVs, recombinant proteins or a combination of OMVs and recombinant proteins (Table 1). Intramuscular (IM) immunizations were done by injecting 1 mL of the vaccine into the muscle on the dorsal side of the neck. Intranasal (IN) immunizations were made with 1 mL volume of the vaccine instilled in one of the nostrils with a plastic Pasteur pipette (Deltalab). Group 1 (control) was administered 1 mL phosphate buffered saline (PBS). Groups 2, 3, 5, 6 and 7 were vaccinated with 30 μg (per protein) of recombinant protein and groups 4–7 were vaccinated with 1.62 × 10^10^ OMV. The OMV dosage was set not to exceed tolerable lipopolysaccharide levels. Groups from 1 to 7 were boosted 4 weeks after the first immunization with a similar vaccine and dose as the first vaccination. Blood was drawn using the vacutainer system with clot activator (Vacutainer) from the jugular vein on day 0, week 2, week 4, week 6 and week 8 after the first vaccination and later, on the day of the *A. pleuropneumoniae* challenge, and three and 7 days post challenge, respectively.

**Table 1:** **Immunization groups**

Group	Number of pigs	Vaccination	Route of immunization
1	4	PBS	IM + IN
2	4	VacJ protein	IM + IN
3	4	ApfA protein	IM + IN
4	4	*A. pleuropneumoniae* OMV	IM + IN
5	4	*A. pleuropneumoniae* OMV + VacJ protein	IM + IN
6	4	*A. pleuropneumoniae* OMV + ApfA protein	IM + IN
7	5	*A. pleuropneumoniae* OMV + VacJ protein + ApfA protein	IM + IN

Immunogen combinations and route of immunization are detailed for each group.

### Challenge

All pigs were challenged with live *A. pleuropneumoniae* HK361 cells [serotype 2; National Collection of Type Cultures (NCTC) 10976], 8 weeks after the first vaccination. *A. pleuropneumoniae* bacteria from an overnight BHI agar culture were incubated in brain-heart infusion (BHI) media supplemented with 5 μg/mL NAD (Sigma Aldrich). When the optical density_600 nm_ (OD_600 nm_) reached 1.5 [corresponding to approximately 2.10^9^ colony-forming units (CFU)/mL according to pilot experiments], the culture was diluted 1:2000 in 30 mL of HEPES saline (10 mM HEPES; 150 mM NaCl, 3 mM KCl, 1 mM CaCl_2_ and 1 mM MgCl_2_) to obtain a concentration of 10^6^ CFU/mL. The diluted solution (10^−1^) was then administered as an aerosol using a NE-U17 OMRON Ultrasonic nebuliser (OMRON Healthcare) and an aerosol chamber where four to five pigs at a time were left for 10 min in a *A. pleuropneumoniae* aerosol. Each chamber session was ended by a period of five min venting, to allow clearing of aerosolised *A. pleuropneumoniae* by passing all air through a HEPA filter before releasing the pigs from the chamber. Pigs were challenged over 2 days, and overall two batches of *A. pleuropneumoniae* were produced and each batch was used for two to three groups of four to five pigs. The initial (pre challenge) *A. pleuropneumoniae* solution (10^−1^) and the leftover solution (post challenge) were plated and incubated on MHF plates in duplicates, for recollection and CFU counting the next day.

### Animal welfare

Clinical signs of the animals were monitored every 3–4 h during the period between challenge and euthanasia. Animals showing signs of distress or a body temperature > 40 °C were administered IM injections of butorphanol tartrate (Torbugesic, Zoetis), dosed according to weight and previous treatment.

### Post-mortem examination

**Figure 2:**
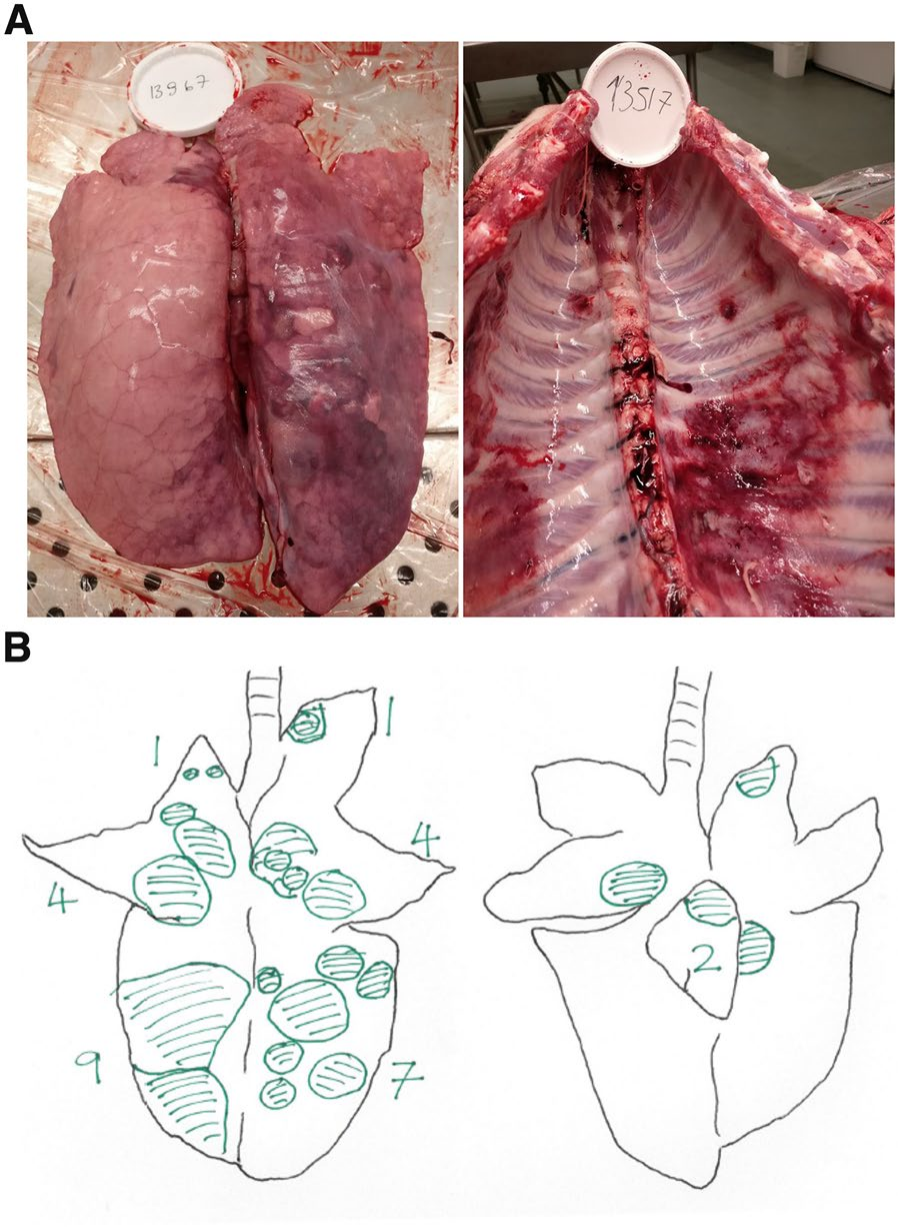
**Scheme and pictures from infected porcine lungs after** ***A. pleuropneumoniae*** **infection.** 7 weeks after their first immunization, pigs were challenged with a concentration of 10^6^ CFU/mL of live *A. pleuropneumoniae* HK361 in an aerosol chamber. 1 week after challenge, pigs were euthanized, and lungs and thorax were examined. **A** Example of lungs and thorax after *A. pleuropneumoniae* infection. **B** Lungs were assessed for their lesion score and number of lesions according to this scheme. Each pair of lungs was assigned a score based on the number and size of the lesions.

Pigs were sacrificed 1 week after the *A. pleuropneumoniae* challenge; lungs were excised from the thorax. Samples from lesions and healthy areas of the lungs were excised and placed in 10% formalin for histological analysis. The pleura and macroscopic lesions of the lungs were assessed systematically using a previously reported scoring system (Figure 2) [42]. Briefly, each of the seven lobes of each lung was assigned a score [0–5] depending on the number and size of lesions. From that the total score of the whole lung was calculated. The statistical significance of the data was assessed using Ordinary One-way ANOVA, Brown–Forsythe, Bartlett’s (*P*  < 0.05) and Dunnett’s multiple comparisons (alpha = 0.05) tests. Correlation between data sets was assessed by Pearson r two-tailed test (alpha = 0.05). After scoring, each lung was weighed and added 1/1 (weight/vol) 0.9% sterile saline solution (Thermo Fisher Scientific) and blended to obtain a homogenized mixture, which was serially diluted tenfold between 1:10^−1^ and 1:10^−7^. 10 μL of each dilution was then plated on MHF plates in duplicates. After overnight (ON) incubation, CFU counts were adjusted in order to reflect the total amount of bacteria contained per gram of lung (CFU/g).

### Antibody titration

Sera were isolated from blood samples by allowing the samples to clot. Nunc Maxisorp flat-bottom 96-wells plates (Thermo Fisher Scientific) were coated with 2 μg/mL ApfA or VacJ proteins, or 3.25 × 10^8^ OMVs in PBS, respectively. Sera from animals in the same group were pooled and added to the wells in three-fold dilutions between 1:500 and 1:1 093 500 (ApfA, VacJ) or 1:1000 and 1:2 187 000 (OMVs). Antibodies specifically recognizing ApfA, VacJ and OMVs were detected with horseradish peroxidase (HRP)-conjugated polyclonal rabbit anti-pig IgG (Thermo Fisher Scientific). Wells were revealed using TMB plus (Kem-En-Tec Diagnostics). Optical density was measured at 450 nm using an enzyme-linked immunosorbent assay (ELISA) plate reader (VersaMax Molecular Devices).

## Results

### Inocula analysis

*Actinobacillus pleuropneumoniae* CFU counts from the inocula are summarized in Table 2. No statistically different CFU count was found between different batches, groups or days of challenge resulting in an expected similar inhaled dose for all pigs.

**Table 2:** **Inocula analysis**

Batch	Administered to group	CFU/mL (pre-challenge)	CFU/mL (post-challenge)
1	1, 2, 3, 4	2 × 10^6^	3.25 × 10^6^
2	5, 6, 7	1.46 × 10^6^	1.85 × 10^6^

Serial dilutions from the inocula before and after challenge were plated on MHF plates. CFU counts are expressed as CFU/mL.

### Histopathological analysis of the lungs

**Figure 3:**
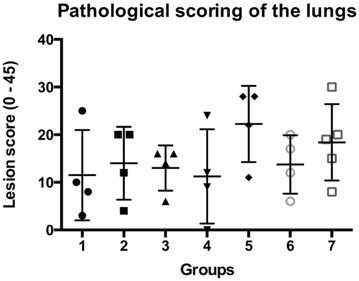
**Lesion scores of the lungs.** Lungs were scored for lesions as previously described [42]. Mean and standard deviation values are graphically reported in figure. Statistical analysis by Ordinary One-way ANOVA, Brown–Forsythe, Bartlett’s and Dunnett’s multiple comparisons tests showed no significant difference between mean scores and standard deviations of each individual group. 1: PBS; 2: VacJ; 3: ApfA; 4: OMVs; 5: VacJ + OMVs; 6: ApfA + OMVs; 7: VacJ + ApfA + OMVs.

**Figure 4:**
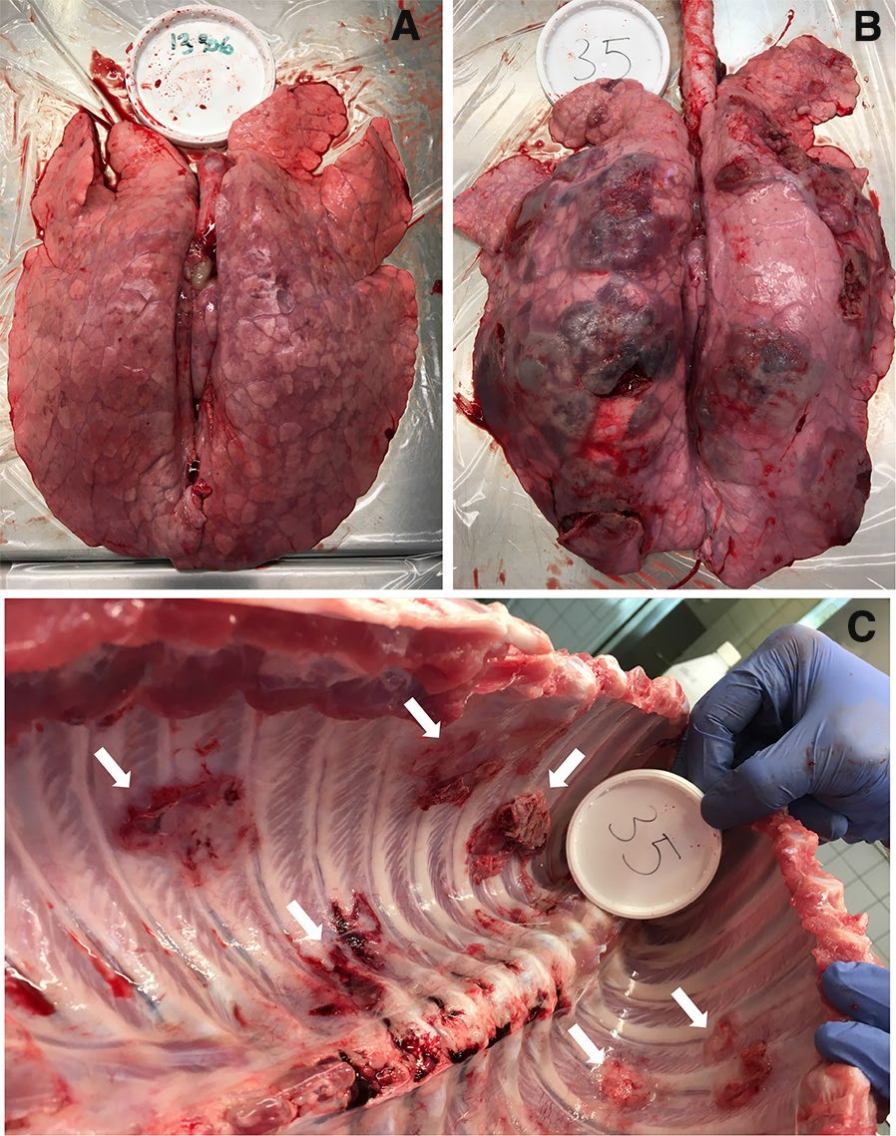
**Post-mortem examination**. Examples of healthy (**A**) and affected lung tissue (**B**) after *A. pleuropneumoniae* infection. Extensive necrosis and purulent lesions are evident in (**B**). **C** Thoracic cavity of the animal whose lungs are portrayed in **B**. White arrows indicate areas where pleuritis caused the adhesion of the lungs to the thoracic cavity.

**Figure 5:**
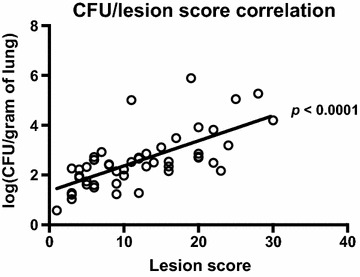
**CFU/Lesion score correlation.** The number of *A. pleuropneumoniae* CFU retrieved from the lungs after euthanasia was plotted against the corresponding lesion score for each individual animal. Statistical analysis by Pearson two-tailed r test showed a significant correlation between number of CFU and lesion score (*P* < 0.0001).

Lesion scores of the lungs are shown in Figure 3. Affected lungs presented lesions from subacute to acute, with signs of multifocal necrosis, fibrinous exudation and purulent inflammation. In some cases the extensive fibrinous exudation to the pleural surface had led to extensive adhesive pleuritis (Figure 4). Some of the most severely affected lungs were partially or completely collapsed, suggesting loss of function (Figure 4). Statistical analysis showed no significant difference between mean lesion scores and standard deviations of each individual group (Figure 3), indicating that none of the vaccine formulations administered were able to successfully prevent *A. pleuropneumoniae* associated colonization and lesions. Instead, statistical analysis by Pearson r two-tailed test showed a correlation between the number of *A. pleuropneumoniae* CFU present in the lungs and lesion scores (*P* < 0.0001) (Figure 5), validating the pathological evaluation performed.

**Figure 6:**
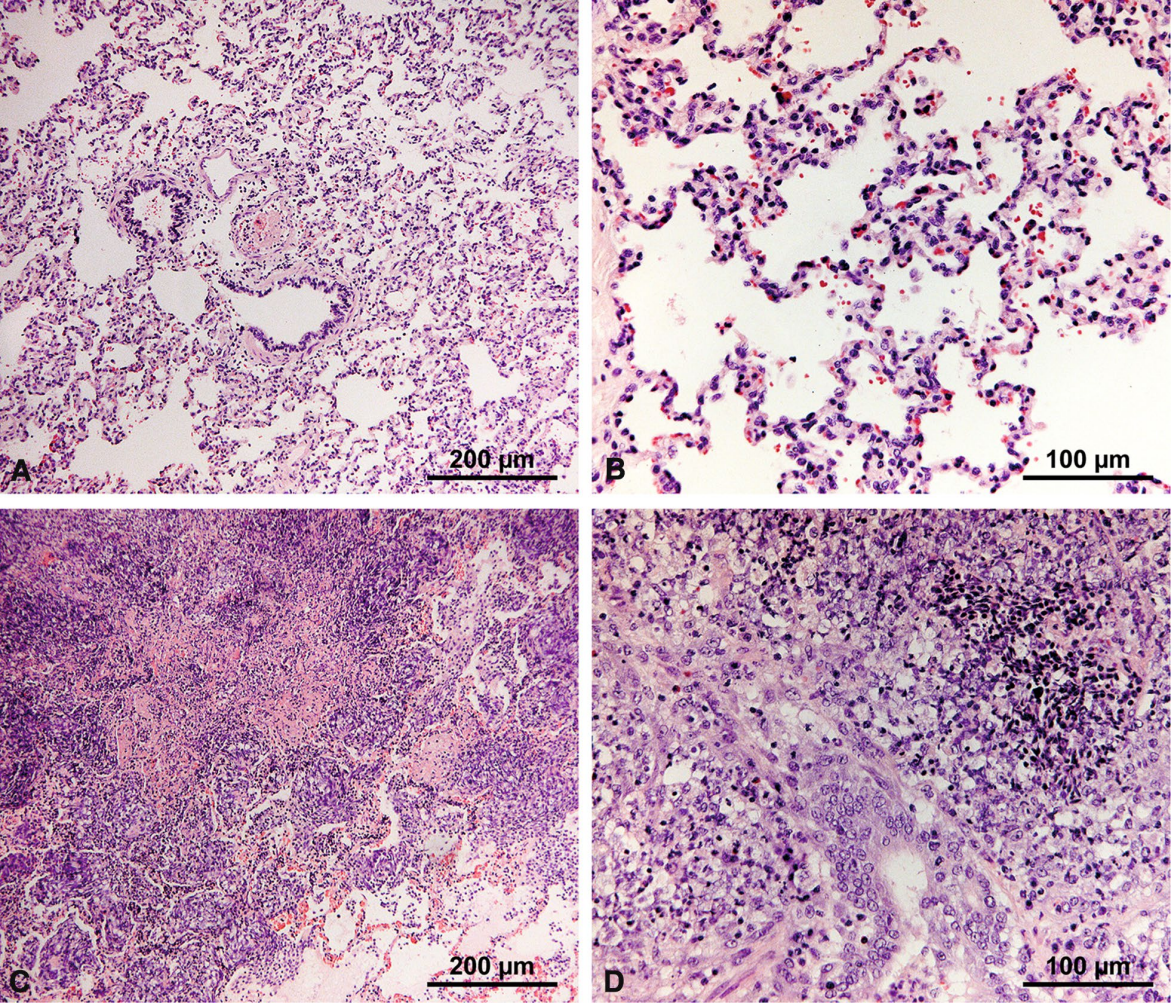
**Histological analysis of the lungs.** Healthy and affected regions of the lungs were cut out and sectioned for histopathological analysis. Sections were stained using hematoxylin and eosin stain (H&E). Only selected representative sections are shown for each type of affected animal. **A**, **B** Sections of healthy regions of the lungs. Normal structure and cellular components of the lungs are visible. **C**, **D** Sections of affected regions of the lungs. Loss of structure and heavy lymphocytic infiltrates are readily appreciable.

Histological analysis of the lesions revealed intense lymphocytic infiltration, with loss of structural integrity and occlusion of the alveoli (Figure 6). No difference could be observed between individual groups, with sections of the lungs similarly affected exhibiting comparable histological characteristics.

### Characterization of IgG response to the immunogens

**Figure 7:**
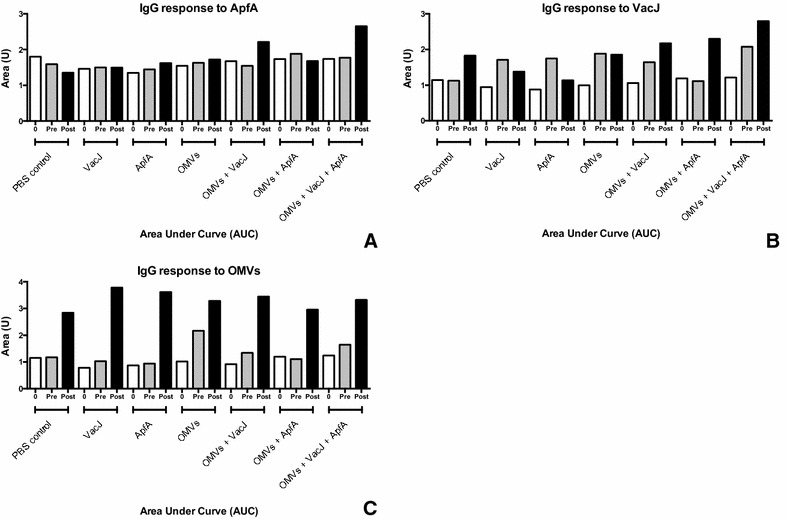
**IgG response to the immunogens.** Sera from animals within each individual group were pooled together and analysed for IgG concentration against ApfA (**A**), VacJ (**B**) and OMVs (**C**). Data are reported as area under curve (AUC), calculated from ELISA titration curves. Three time points are shown: 0: first immunization; pre: second immunization; post: endpoint.

IgG titers from pig sera are shown in Figure 7. The sera retrieved from animals belonging to the same group were pooled together for immunological analysis. As such, the serological data and the resulting conclusions presented in this study are to be considered representative of trends per group rather than per individual animal.

**Figure 8:**
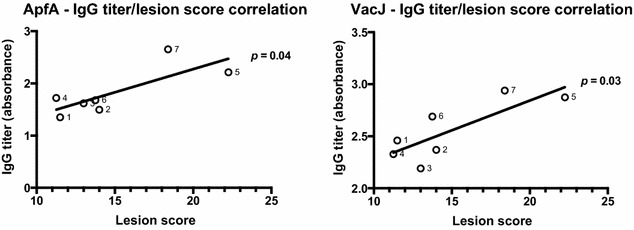
**IgG titer/lesion score correlation.** Combined IgG titers for each animal group (1–7) against ApfA (**A**) and VacJ (**B**) were plotted against the average lesion score of each individual group. Pooled endpoint sera retrieved on the day of euthanasia (1 week after challenge) were used. Statistical analysis by Pearson two-tailed r test showed a significant correlation between average IgG titers and lesion scores (ApfA *P* = 0.04; VacJ *P* = 0.03).

Immunization with ApfA and VacJ proteins alone did not produce an appreciable increase in specific IgG antibodies against the proteins (Figure 7), suggesting the ineffectiveness of immunizing with these proteins individually. Furthermore, immunization with ApfA induced an IgG response against VacJ in the sera of vaccinated animals, suggesting that ApfA may interfere with the development of immunity against co-administered immunogens, as reported before [43]. Interestingly, the animals in the control group presented a rather high titer against VacJ after challenge, pointing to the in vivo immunogenicity of this protein (Figure 7). As expected given their multi-antigenic nature, the overall antibody response to the OMVs was conspicuous in all groups, with the highest titers reached when the OMVs were administered alone (Figure 7). Administration in combination with either of the other immunogens decreased the response to the OMVs, particularly before challenge, increasing on the other hand the response to the other immunogens (Figure 7). This was especially true in the case of VacJ, were groups that received both VacJ and OMVs showed steadily increasing and overall high IgG titers, while groups that received only VacJ showed a decrease in titer, even after challenge (Figure 7). Furthermore, statistical analysis by Pearson r two-tailed test showed a positive correlation between IgG titers elicited against the ApfA and VacJ immunogens and lesion scores (ApfA *P* = 0.04; VacJ *P* = 0.03), suggesting a major negative role of the immune system in *A. pleuropneumoniae* pathogenesis (Figure 8). No correlation was observed between IgG response to the OMVs and lesion scores.

## Discussion

Effective prevention of *A. pleuropneumoniae* outbreaks has remained elusive for decades, with available vaccines unable to confer cross-serotype protection or prevent lung colonization by *A. pleuropneumoniae* cells [11, 13]. To date, the most effective strategy relies on targeting a family of toxins centrally involved in *A. pleuropneumoniae* pathogenesis, the RTX toxins [22, 23]. Vaccines containing RTX toxoids have been shown to effectively prevent the clinical manifestations of the infection [7]. Unfortunately, this approach comes short of stopping lung colonization, and animals immunized in this way have been showed to be possible *A. pleuropneumoniae* carriers [44]. For this reason we chose to use different combinations of OM proteins and OMVs as immunization strategies, similarly in principle to how the widely used Bexsero^®^ vaccine against *Neisseria meningitidis* was conceived [45].

Our data showed that none of the immunization strategies employed was able to successfully prevent *A. pleuropneumoniae* colonization or the development of lesions associated with infection. This may be due to different reasons, and our data, together with previously published studies [11, 25, 35], suggest caution in dismissing the selected immunogens as simply ineffective for immunization. As already mentioned, *A. pleuropneumoniae* possess several resistance mechanisms that allow it to avoid clearance by the host immune system. Accordingly, it is possible that raising antibodies against valid OM antigenic targets may not be sufficient for the host immune system to clear the infection before the RTX toxins and other virulence factors start to be released. Once released, most of *A. pleuropneumoniae* toxins can target specifically lymphocytes, and thus kill a wide range of the cells involved in clearance [23]. If this is the case, we hypothesise that a combination of OM immunogens and RTX toxoids may represent a valid immunization strategy for the prevention of both colonization and pathogenesis by *A. pleuropneumoniae*.

Another possibility comes from the role played by the host immune system during *A. pleuropneumoniae* infections. *A. pleuropneumoniae* cells are able to induce the release of pro-inflammatory mediators by activated phagocytes during infection, leading to extensive cytotoxicity due to the production of oxygen radicals and proteases [3, 22, 23]. Here we described a correlation between IgG titers against ApfA and VacJ immunogens and lesion scores (Figure 8), suggesting that a different cytotoxic pathway may be involved in the development of the lesions associated with *A. pleuropneumoniae*. This alternative pathway is known as antibody-dependent cell-mediated cytotoxicity (ADCC), and has been demonstrated to be involved in the pathogenesis of several bacterial infections [46, 47]. Our data point to the involvement of ADCC in *A. pleuropneumoniae* infections, showing that the groups with the highest IgG titers were the ones that had the highest lesion scores (Figure 8). Furthermore, the histological analysis of the lungs seemed to confirm a major role for the host immune system in the development of the lesions, in which affected lungs were heavily infiltrated by lymphocytes and showing extensive fibrinous exudation on occasions (Figures 4 and 6). This hypothesis may also explain the high variability in lesion score observed between animals belonging to the same group, as the pathological outcome of an *A. pleuropneumoniae* infection could be much more dependent on the individually variable immune response than on infectious dose (ID). Although the role of ADCC in App pathogenicity has yet to be proven, it has been shown that a high seroconversion rate towards the PalA protein produced by App is inversely correlated with protection level in animals immunised with this protein [48]. Notably, no correlation was present between IgG response to the OMVs and lesion scores, likely due to the generalised high response in all animals to the LPS present in the OMVs. Indeed, if ADCC is effectively responsible for considerable damage in *A. pleuropneumoniae* infections; we could be forced to rethink our approach to both treatment and prevention, as raising IgG titers against *A. pleuropneumoniae* OM antigens may prove counterproductive. In this scenario, a combination of immunization by RTX toxoids and a desensitisation regime toward ADCC could achieve better results and be instrumental in reducing antibiotic consumption.

As expected, immunization with the OMVs elicited a rather high IgG titer in the animals, exhibiting adjuvant properties when administered in combination with the other immunogens (Figure 7). This did not come as a surprise, as the adjuvant potential of the OMVs has been widely demonstrated in previous studies [29, 49, 50]. On the other hand, ApfA and VacJ elicited a rather low response when administered alone (Figure 7). Our results contrast with previous reports on ApfA, indicating both immunogenic and protective potential in vivo [11, 26]. Interestingly, VacJ was found to elicit a consistently high IgG titer after challenge (Figure 7), suggesting that this protein may possess a relevant immunogenic potential in vivo.

Finally, our results show a general reliability of the challenge model used and the effectiveness of the aerosol chamber as a biologically relevant method to study the pathogenesis of *A. pleuropneumoniae*. OMV dosage during immunization was effective in eliciting the maximum immune response within tolerable parameters of toxicity. All animals received approximately the same amount of *A. pleuropneumoniae* CFU during challenge and developed a subacute infection, while none of them needed to be euthanized before the planned endpoint due to critical clinical conditions. Moreover, the positive correlation between CFU in the lungs and lesion score shows the validity of the scoring system used.

In conclusion, our study provided new insight into *A. pleuropneumoniae* pathogenicity and the role played by host-derived cytotoxicity. The aerosol chamber represented a reliable and effective device for *A. pleuropneumoniae* challenge, ensuring that all animals were exposed to the same ID through the natural route of infection. On the other hand, the evident contrast between the overall technical reproducibility achieved and the high variability in lesion score within each group underlines the complexity of the *A. pleuropneumoniae* pathogenesis. To address this issue and provide a more solid statistical basis, larger groups of animals may be needed during similar *A. pleuropneumoniae* trials in the future.

## Abbreviations

*A. pleuropneumoniae*: *Actinobacillus pleuropneumoniae*
OM: outer membrane
OMV: outer membrane vesicles
NAD: nicotinamide dinucleotide
*E. coli*: *Escherichia coli*
PBS: phosphate buffered saline
IM: intramuscular
IN: intranasal
BHI: brain–heart infusion
NCTC: National Collection of Type Cultures
OD_600 nm_: optical density_600 nm_
CFU: colony-forming units
ON: overnight
HRP: horseradish peroxidase
ELISA: enzyme-linked immunosorbent assay
ADCC: antibody-dependent cell-mediated cytotoxicity
ID: infectious dose
